# Enhancement of electrical conductivity associated with non-bridged oxygen defects in molybdenum phosphate oxide glass via doping of SrO

**DOI:** 10.1038/s41598-023-45333-7

**Published:** 2023-10-26

**Authors:** A. M. Fayad, M. A. Ouis, R. M. M. Morsi, R. L. Elwan

**Affiliations:** 1 Glass Research Department, National Research Centre, Dokki, Giza, Egypt; 2 Physical Chemistry Department, National Research Centre, 33 El Bohouth St., Dokki, Giza, 12622 Egypt

**Keywords:** Energy science and technology, Materials science

## Abstract

Based on the attractive properties of phosphate glass, improved molybdenum phosphate glasses of composition 40P_2_O_5_, 20MoO_3_, 15MgO, (25-x)Li_2_O, xSrO, [x = 0, 5, 10, 15 and 20 mol %] were prepared via the melt**-**quench technique. They were characterized by X-ray diffraction (XRD), Fourier transform infrared spectroscopy (FTIR), UV–visible reflectance and Electron spin resonance (ESR). FTIR confirmed the existence of several structural phosphate groups other than MoO_4_ and MoO_6_ units. Optical analysis revealed the active species of molybdenum ions. SrO addition decreases the bandgap energy, converting the glass insulator features into semiconductor properties. The measured AC electrical conductivity (σ_ac_) increased within the temperature range of 298–473(K) and decreased in the frequency range of 0.042 kHz–1 MHz. The estimated DC electrical conductivity increased with temperature, suggesting the semiconducting behavior. The highest electrical conductivity was found in base and 5% SrO samples. Therefore, it appears that the prepared glasses are viable candidates for opto-electronic applications.

## Introduction

Phosphate glasses excel with desirable properties such as a low melting point, high thermal expansion coefficient, and high ultraviolet transmission in contrast to common oxide glasses^1–3^. The open structure of the phosphate chain allows incorporating property modifiers such as heavy metals, transition or rare earth elements which greatly improve the glass's thermal stability and toughness as well as its chemical resistance. Phosphate glasses have achieved unique magnetic, optical, and electrical properties which meet the requirements of many modern applications^1–3^. Recently, phosphate glasses are considered promising as new solid-state electrolytes owing to their structure that permits a high mobility for protons^4,5^.

Many prior studies on phosphate glass have focused on the effect of both alkalis and alkaline earth oxides on its structure and properties in the context of improving its optical, physical, and electrical qualities^6–8^. Li_2_O-P_2_O_5_ glass was found to be an outstanding fast-conducting glass for Li^+^ ions. Depending on the concentration of Li_2_O, the addition of Li_2_O can improve phosphate glass's ionic conductivity^6,7^. Strontium oxide was also used to improve the durability of phosphate glass^9,10^. SrO may be useful in improving the electrical conductivity of phosphate glasses. Furthermore, Bouabdalli et al.^10^ Found that adding SrCO_3_ and Li_2_CO_3_ to the boro-phosphate matrix reduces the phonon energy via the exchange of BO_4_ ↔ BO_3_ and PO_3_ ↔ PO_2_, resulting in improved fluorescence efficiency and a longer lifetime.

MgO is utilized as a glass modifier. Its molecules break the random glass network creating bonding defects which result in free paths^8,11^. Because lithium ions are light and tiny, they can move freely across the glass network. The ionic conductivity of glass is affected by this phenomenon. Mg^2+^ ions occupy the glass system's interstitial sites and disintegrate the P-O-P bonds, generating bonding deformations. This process leads to empty cavities and easy tracking by facilitating the doped ions' movement^8,11^. As a result, MgO plays a crucial role in improving the samples' dielectric features.

Molybdenum oxide and P_2_O_5_ combine to generate binary glasses with a broad and continuous glass-forming range. The structure of molybdenum phosphate glass systems has received a lot of attention^12,13^. The presence of molybdenum ions in two different valence states Mo^5+^ (which occupy octahedral sites) and Mo^6+^ (which occupy tetrahedral and octahedral sites) was identified, and the redox [Mo^5+^/Mo^6+^] ratio increased with MoO_3_ concentration increase^12,13^. Molybdenum's six-coordinated environment was further identified in the Na_2_O-MoO_3_-P_2_O_5_ glass structure^14^. The electrical properties of sodium and lithium phosphate glasses were studied by impedance spectroscopy to evaluate the electronic and ionic contributions to conduction. It indicated that there is no correlation between the variation in electronic or ionic conductivity and the maximum Mo^5+^/Mo_tot_ and Mo^5+^/Mo^6+^ ratio in these glasses^14,15^. An optical absorption investigation shows that increasing the MoO_3_ content up to 0.3 mol% results in the conversion of molybdenum ions from tetrahedral sites to octahedral sites. While the concentration of molybdenum ions that exceed 0.3 mol% assists in glass network formation^16^. Molybdenum phosphate glasses have received major concern for their ionic and electronic transport properties^2,17,18^. As known, pure ionic-conducting glasses can be used as solid electrolytes in electrochemical cells, whilst mixed ionic-electronic glass conductors have the potential to be useful as cathode materials^19^.

Optical and electrical properties are suitable for describing glass structure as a function of its composition and network connectivity. Previous literature indicated that reports on optical and electrical properties of glasses containing combined SrO and Li_2_O are limited. To the best of our knowledge, ternary P_2_O_5_, MoO_3_, and MgO glass systems doped with both SrO and Li_2_O have not been investigated.

In the current work, the structural properties of studied glasses in the system 40 P_2_O_5_, 20 MoO_3_, 15 MgO, (25-x) Li_2_O, x SrO where 0 ≤ x ≤ 20 mol% are examined based on XRD analysis, density measurements, molar volume calculations, hardness and FTIR spectroscopy. The optical properties as a function of structural changes induced by SrO addition are assessed. Moreover, the effect of SrO concentration on the electrical properties of these glasses has been reported, which may be suitable for use in opto-electronic devices.

## Experimental

### Glass preparation

The glass samples were obtained by melting a mixture of calculated proportions of pure materials NH_4_H_2_PO_4_, SrCO_3_, Li_2_CO_3_, MoO_3_, MgO in porcelain crucibles in a SiC-heated furnace (Vecstar, UK) at 1200 °C for 2 h with repeated rotation of the melts to ensure perfect blending and homogeneity. The completed molten of each sample was cast into warmed stainless-steel molds to get the desired dimensions (2 cm × 1cm). These samples were then transferred to a regulated muffle furnace at 320 °C for annealing. Keeping the samples in the furnace for 1 h, the furnace was switched off and allowed to cool at 30 °C/h to room temperature.

### XRD

X-ray diffraction (XRD) was measured on fine powders of the obtained samples. Samples were ground and the fine powder was examined using a diffractometer adopting Ni-filter and Cu-target. The X-ray diffraction patterns were obtained using a Philips PW 1390 X-ray diffractometer. The diffraction patterns were identified with comparison with standard ASTM cards and published related data. The diffractometer with Cu radiation (λ = 1.5405 Å) operating at 40 kV, 30 mA, at room temperature was used. The acquired diffraction patterns are ranging from 5° to 80° for 2θ values.

### Density and molar volume, microhardness

Density of glasses was measured at room temperature, using the suspended weight method based on Archimedes principle according to the following formula,

1 $$\uprho \, = \, \left[ {{\text{ a}}/\left( {{\text{a}} - {\text{b}}} \right)} \right] \, \times \, 0.{86 } = {\text{ g/cm}}^{{3}}$$

where ρ is the density of the glass sample, a and b are the weights of the glasses in the air and in xylene respectively, and 0.86 is the density of xylene at 20 °C. Molar volume of the glasses was calculated using the following formula:

2 $${\text{Vm}} = {\text{ M}}/\uprho \, = {\text{ m}}^{{3}} /{\text{mol}}$$

where, M is the molar mass of elements in each glass sample individually, and ρ is the glass density. The specific volume of the glasses was calculated by using the inverse formula of density values.

The hardness was measured by using a Knoop diamond (KHN), under a load of 25 g, applied for 10 s (HMV microhardness tester; Shimadzu Type-M Tokyo, Japan). Three sequences of five indentations were performed.

### FTIR

Fourier transform infrared spectroscopy was measured by (FT-IR- Brucker Vertex 8V, Germany), in the range of 1600 to 400 cm^−1^ wavenumbers with a resolution of 4 cm^−1^, to identify the structural network groupings.

### Optical measurements

The diffuse reflectance spectra (DR) of the glass powders were recorded within the range of 200–1750 nm utilizing a UV–Vis near-infrared spectrometer (UV-3150, Shimadzu, Tokyo, Japan) equipped with the ISR-3100 integrated sphere. Spectra were measured at room temperature with a 0.3-nm resolution. The Kubelka–Munk function F(R) is directly proportional to absorbance. Hence, F(R) values were altered to the linear absorption coefficient α using the next relation:

3 $$\upalpha \, = {\text{ F}}\left( {\text{R}} \right)/{\text{t}}$$

where t is the thickness of the sample.

In the limiting case of infinitely thick samples, neither thickness nor sample holder affect reflectance (R). In this case, the Kubelka–Munk equation at any wavelength becomes the following^20,21^:

4 $${\text{F }}\left( {\text{R}} \right) = \left( {{1} - {\text{ R }}} \right)^{{2}} /{\text{2R}}$$

where R = R_sample_/R_standard_. As a result, the following relational expression was applied as proposed by Tauc et al.^20,21^.

5 $$\upalpha {\text{hv }} = {\text{ A}}_{{1}} \left( {{\text{hv }} - {\text{ E}}_{{\text{g}}} } \right)^{{\text{n}}}$$

where h is Planck's constant, ν is frequency of vibration, α is absorption coefficient, E_g_ is band gap n is the type of transition (1/2 in the case of direct transition or 2 in the case of indirect transition) and A_1_ is proportional constant.

Diffuse reflectance spectra were transformed to the Kubelka–Munk function. Thus, the vertical axis became F(R), whose value corresponded to the absorption coefficient. Then α in the Tauc equation, it is replaced with F(R). So, in the actual experiment the relational expression develops into^22,23^:

6 $$\left[ {{\text{F}}\left( {\text{R}} \right){\text{hv}}} \right]^{{{1}/{\text{n}}}} = {\text{ A}}_{{2}} \left( {{\text{hv }} - {\text{ Eg}}} \right)$$

Consequently, getting F(R) from Eq. (4) and plotting the [F(R)hν]^1/n^ against hν, the band gap E_g_ of powder samples can be obtained simply, where A_2_ is proportional constant.

### ESR

The prepared glasses' ESR spectra were measured using an EMX spectrometer (X-band). The cavity selected was a standard Bruker ER 4102 rectangular cavity, Germany. The operating parameters were as follows: microwave power 0.796 mV, modulation amplitude 5 Gauss, time constant 81.92 ms and conversion time 20.48 ms. The powder of samples was inserted into ESR tubes and measured at the mentioned parameters. All ESR measurements were performed at 25 °C during a single scan.

### Electrical measurements

The AC conductivity (σ_ac_) of the prepared samples was measured with LRC Hi-Tester (HIOKI, 3532-50), Japan, in a frequency range from 0.042 kHz to 1 MHz, and at temperatures ranging from 298 to 473 K. Temperature rising was achieved via increasing the input voltage from a variac transformer connected to a wire-wound resistance heater. In close proximity to the sample, a copper/constantan thermocouple was used to determine the temperature. The capacitance, C, and the dissipation factor, tan δ, are acquired instantly from the instrument for the samples. The AC conductivity, σ_ac_, is calculated using the relation:

7 $$\upsigma_{{{\text{ac}}}} = \, \upomega \varepsilon_{o} \varepsilon \prime {\text{ tan }}\updelta ,$$

where ω is the angular frequency, ε_ο_ is the free‐space permittivity and ε′ is the dielectric constant which is determined from the expression

8 $$\varepsilon \prime \, = {\text{ C d}}/\varepsilon_{o} {\text{A}},$$

where d is the thickness of the sample and A is the cross‐sectional area^24^.

## Results and discussion

### X-ray diffraction analysis (XRD)

**Figure 1:**
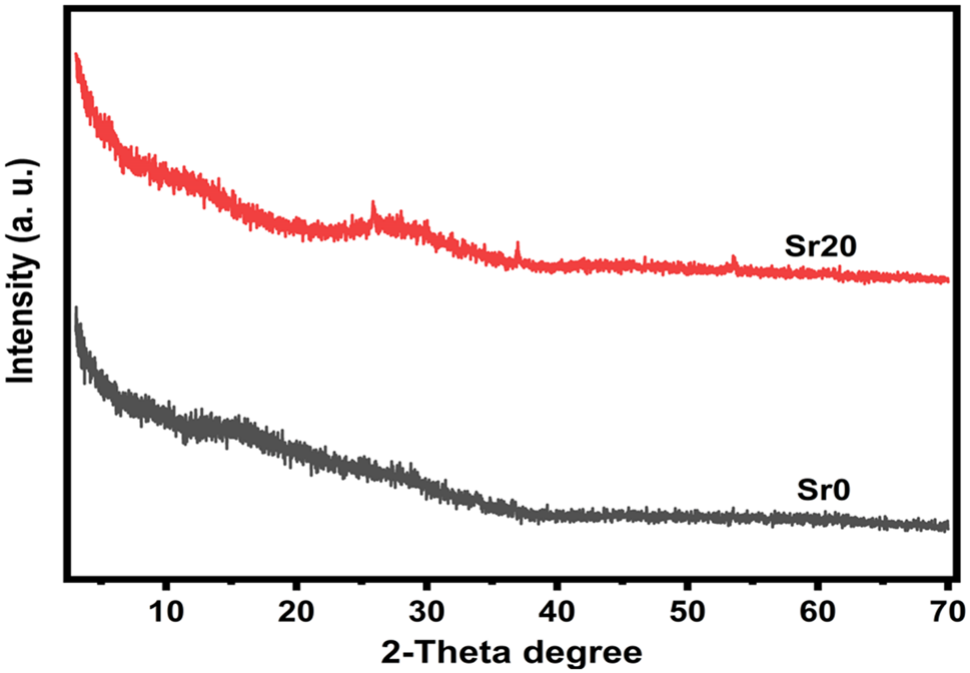
X-ray pattern of samples Sr0 and Sr20.

XRD is a crucial experimental method for determining structure, grain size, lattice strains, and other aspects of material characterization. The produced samples lack sharp peaks and are amorphous^25^ and are verified to be non-crystalline. All produced samples' XRD patterns support amorphicity as depicted in Fig. 1.

### Density, molar volume and microhardness

Density measurement has been extensively studied to understand how composition affects glass structure. The network compactness, change in geometrical arrangement, cross-link density, and molar mass of the components are the primary determinants of glass density^26^.

**Figure 2:**
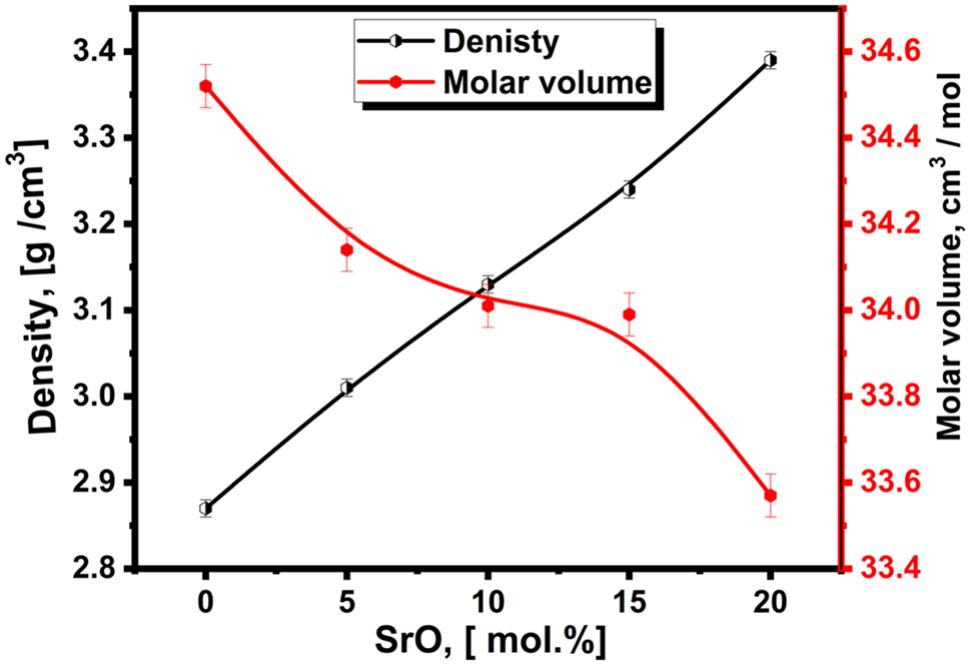
Density and molar volume of prepared samples.

The variation in measured density and calculated molar volume values of the glass samples is shown in Fig. 2, Table 1. It is observed that the successive partial substitution of Li_2_O with SrO results in a rearrangement and change in the geometrical configuration of the atoms in the host glass lattice. The density increased gradually up to 20 mol% SrO while the molar volume decreased as increasing SrO oxide content replacing Li_2_O oxide. The density increase might be explained by the fact that Li has a smaller molar mass (6.941 g/mol) than Sr (87.6200 g/mol). From another standpoint, SrO (4.7 g cm^−3^) has a heavier density than Li_2_O (2.01 g cm^−3^). Furthermore, the density increase (decrease in molar volume) was accompanied by the existence of varying structural units of PO_2_, PO_3_, and PO_4_. The conversion of some MoO_6_ into MoO_4_ and the potential increase in non-bridging oxygen support the increase in density.

**Table 1:** The chemical composition and some physical properties of studied glasses.

Glass no.	Glass composition mol%					Density (ρ) (g/cm^3^) ± 0.01	Molar volume (V_m_) (cm^3^/mole) ± 0.05	Vickers microhardness (H_v_) (kg/mm^2^) ± 3
	P_2_O_5_	MoO_3_	MgO	Li_2_O	SrO			
Sr0	40	20	15	25	0	2.87	34.52	280.25
Sr5	40	20	15	20	5	3.01	34.14	286.33
Sr10	40	20	15	15	10	3.13	34.01	414.25
Sr15	40	20	15	10	15	3.24	33.99	487
Sr20	40	20	15	5	20	3.39	33.57	641.75

Glass hardness is one of its most crucial qualities since it gauges how dense the structure is. Microhardness values show how different cation substitutions affect glass. Table 1 lists the values. The microhardness values of glass samples were raised when Li_2_O was replaced with SrO leading to an increase in glass network coherence. The increased glass microhardness may be attributed to the strontium-oxygen connection strength being higher than that of the lithium-oxygen link^27^. As known, Sr^2+^ has a stronger cationic field strength (0.29 A^−2^) than Li^+^ (0.26 A^−2^)^27,28^.

### FTIR studies

**Figure 3:**
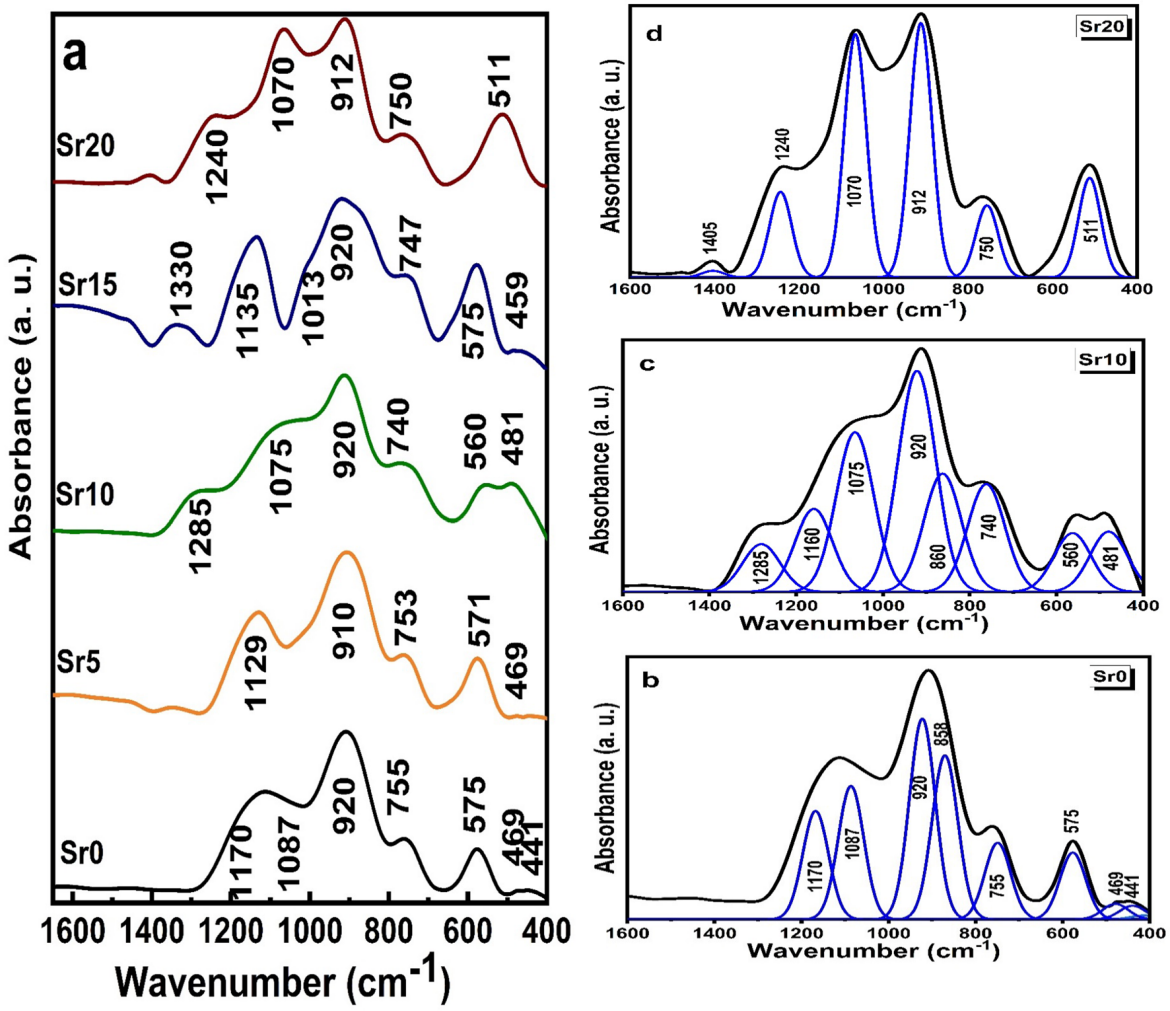
FTIR absorption spectra of (**a**) prepared samples and (**b**–**d**) the deconvolution spectra of some Sr0, Sr10 and Sr20 samples.

Oxide glass FTIR investigations provide information on structure as well as the compound's oxygen coordination number, network formers, and change in oxygen bonds of the framework that is also produced by the cation modifiers. Figure 3 demonstrates the FTIR absorption spectra of the current series of molybdenum phosphate glass samples that are doped with successive increasing SrO content on expense of Li_2_O. It is reasonable that vibrational signals originate mostly from the phosphate matrix as it is the most predominant composition. The addition of alkali or divalent oxides induces linear chain structure of the phosphate molecule^29^. This structure is created by cleaving P–O–P bonds and creating non-bridged oxygens (NBOs). These divalent ions act as ionic crosslinks among NBOs of two different chains. This action is typically carried out by alkaline cations such as (Ca^2+^, Sr^2+^, Ba^2+^). But there are some divalent oxides such as (ZnO, MgO) form structural units (ZnO_4_, MgO_4_). As mentioned, molybdenum ions get into the glass network as a former and/or modifier when bound to four or six oxygen ions to form tetrahedral coordinated MoO_4_ and octahedral coordinated MoO_6_ units. Molybdate structural units (isolated MoO_4_) revealed infrared absorption at wavenumbers 920–900, 850–830, 800 and 430–410 cm^−1^ as established in previous published data^30^. Moreover, symmetric and asymmetric vibrations of Mo–O–Mo links can be noticed near 600 and 450 cm^−1^, respectively. Accordingly, FTIR infrared absorption spectra measurements are expected to show a clearer image of the structural lattice of the investigated phosphate glasses with varied modifier cations. The phosphate species in phosphate glasses mainly occur within the wavenumber range 1600–400 cm^−1^. Considering the deconvoluted FT-IR data, the current glass network has a broad spectrum of IR-active bands. Based on the literature the origin of the identified bands can be explained as follows^29–36^.

- The absorption peaks in the wavenumber region below 600 cm^−1^ are related to the vibrations of modifying cations (such as Ca^+^, Sr^2+^, Na^+^ and Li^+^) at different interstitial locations. The band around 469 cm^−1^ corresponds to the bending oscillations of O–P–O groups, δ (PO_2_) modes of (PO_2_^−^)_n_ chain units. In addition, symmetric and asymmetric Mo–O–Mo links vibration, vibrations of Mg^2+^ cations in their sites.
- The presence of the absorption band at 575 cm^−1^ correlates with the fundamental frequency of PO_4_^–3^ or to P=O bending vibrations. The existing mode at 755 cm^−1^ is ascribed to υ_s_ (P–O–P) linkages in the pyrophosphate group (P_2_O_7_)^4−^, while those around 920 and 858 cm^−1^ are accredited to υ_as_ (P–O–P) linkages besides isolated MoO_4_.
- The bands around 1087 and 1170 cm^−1^ are determined from asymmetric stretching vibration of the (PO_3_)^2−^ terminal group, υ_as_ (PO_3_)^2−^.

It was observed that the location, widening and intensity of absorption bands are affected by Sr ions’ entrance. The band at 441 cm^−1^ disappeared with Li_2_O decrease. The peaks in the wavenumber range 400–580 cm^−1^ became one medium broad peak at wavenumber around 511 cm^−1^ by SrO addition. Moreover, vibrations spanning between 1000 and 1300 cm^−1^ and the disappearance of the band at 850–860 cm^−1^ illustrate the modification behavior of SrO in the phosphate lattice. This is done through the formation of different anionic groups or NBOs. These groups are categorized as PO_2_^−^, PO_3_^2−^, and PO_4_^3−^, found in Q_2_, Q_1_, and Q_0_, respectively^35,36^. The observed peaks at 1240-1290 and 1013 cm^−1^ with different SrO contents are ascribed to asymmetric stretching oscillations of O-P-O links of (PO_2_^−^)_as_ and (PO_3_^2−^)_as_ located in Q_2_ and Q_1_ units, respectively^35,36^. Thus, all the vibration bands detected in the infrared spectrum are within the framework of the modification of the molybdenum phosphate glass network. This modification is associated with the strontium oxide content.

### Optical properties

**Figure 4:**
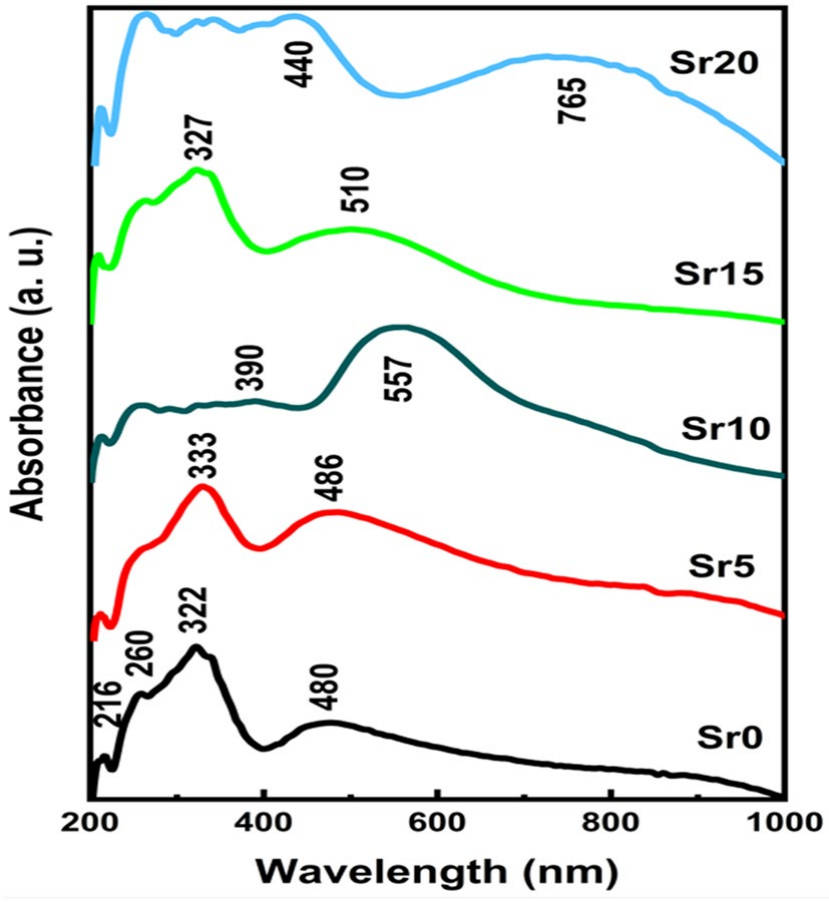
The normalized absorption spectra of prepared glasses.

The normalized absorption spectra of 40% P_2_O_5_, 20% MoO_3_, 15% MgO, (25-x) Li_2_O, and x % SrO are shown in Fig. 4. Based on the Kubelka–Munk function, these spectra were transformed from diffuse reflectance spectra as clarified elsewhere^23^. Optical absorption data indicate interesting variations upon continuous substitution of specific ratios of Li_2_O with SrO. This is correlated to the strong effect of the existing alkaline oxides, magnesium, and strontium oxide on the optical characteristics of the studied glass lattice. Whereas the incorporation of alkaline earth elements into the glass matrix depolymerizes the glass network then stimulates the formation of non-bridging oxygen species (NBO). Furthermore, the presence of MgO and successive addition of SrO at expense of Li_2_O, is expected to cause variations in the ligand field around the Molybdenum probe ion owing to alterations in the oxygen ion polarizability that surrounding molybdenum ion as well as its reliance on the field strength of the glass matrix^37,38^. Otherwise, some previous studies^39–41^ focused on UV–visible investigations of molybdenum ions in glass. They realized that it is acceptable for molybdenum ions to exist in the glass matrix as either trivalent, tetravalent, pentavalent, hexavalent, or a mixture of some of them. Weyl^42^ attributed the green color of phosphate glass to pentavalent molybdenum ions under severely reducing conditions and suggest that the Mo-doped glasses display blue-green, green, brown, violet, and faint yellow resulting from Mo^6+^, Mo^5+^, Mo^4+^, and Mo^3+^ ions. ElBatal et al.^42,43^ and others^34,44^ investigated the structure and optical features of some phosphate glasses doped with MoO_3_. They reached to that the presence of Mo^6+^, Mo^5+^, Mo^4+^ and Mo^3+^ ions together within the studied glasses. In addition, each valence state of molybdenum ions is characterized by a specific visible absorption band. Mo^3+^ and Mo^5+^ions display absorption bands at 350–370 nm and 420–450 nm, Mo^4+^ ions are assigned at 550–580 nm beside the visible absorption in the wavelength range 700–960 nm is correlated to Mo^5+^ ions.

As depicted in Fig. 4 the present glasses reveal an appearance of UV–visible absorption bands with some changes associated with the consecutive substitution of Li_2_O with SrO. The first sample with 0 SrO content shows UV-near visible absorption peaks at about 216, 260, and 322 nm accompanied by a visible absorption peak at 480 nm plus weak broad absorption in the 800–1000 nm wavelength range. The UV-near visible spectrum range in the glass samples with 10–20 mol% SrO extended and show extra absorption peaks at 346 and 390 nm. With SrO content increases the absorption intensity of the band at 480 nm increases and shifts to higher wavelength at 557 and 510 nm in 10% and 15% SrO. The broad visible absorption at 800–1000 nm reached the highest intensity and shifted to lower wavelength centered at 765 nm in the highest SrO content glass sample. The identified absorption in UV region could be clarified as it was studied by many researchers, and they attribute it to trace iron impurities (Fe^3+^) that are indeed contaminate the raw materials^45,46^. Urbach^47^ attributes this UV absorption edge to electron transitions between oxygen 2p and transition metal ion 3d states. It is noteworthy, the highest intensity of the broad band observed in the spectrum of glass sample that contains 20% SrO suggests that this glass includes the highest concentration of Mo^5+^ ions. It is expected that such Mo^5+^ ions will form Mo^5+^O^3−^ molecular orbital states and take part in the depolymerization of the glass matrix^44,48^, leading to more bonding defects and non-bridged oxygens (NBO’s). It is obvious that glass samples' absorption spectra comprise expanded UV-near visible peaks alongside the observed wide peak centered at about 760 nm. It is clear from this that the investigated molybdenum phosphate glasses reveal absorption bands which elucidate that the valences of molybdenum ions (Mo^3+^, Mo^4+^, Mo^5+^ and Mo^6+^) substantially exist with varied ratios. While the hexavalent state of Mo ions (d^0^) exhibit UV band that may interfere with strong charge transfer peaks due to trace iron contaminants, it cannot be excluded^39^.

**Figure 5:**
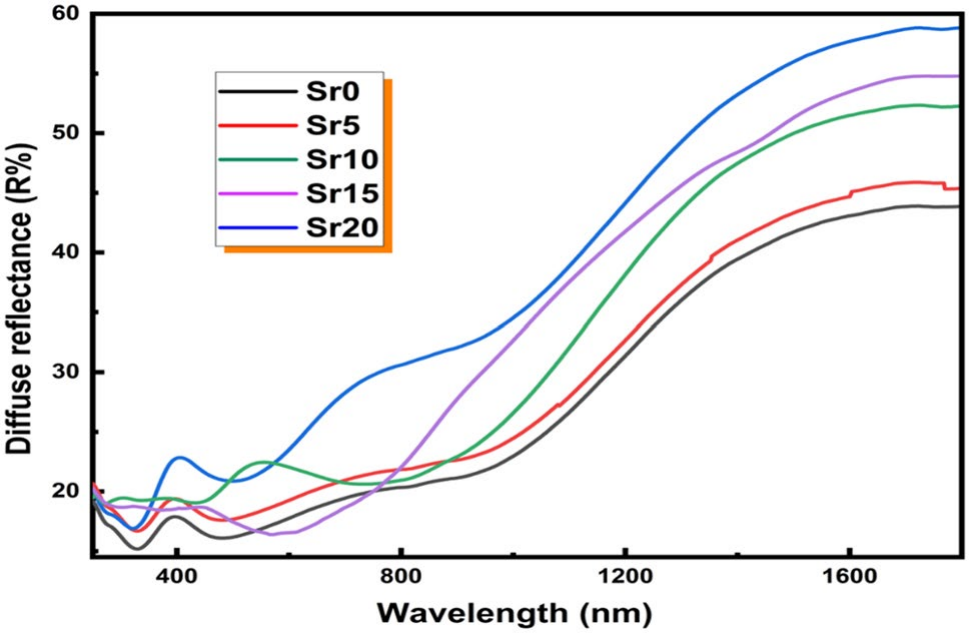
The diffuse reflectance (DR) spectra of the prepared glasses.

Diffuse reflectance spectroscopy, especially the bandgap energy, is valuable for probing optically induced transitions. Furthermore, it provides information on the structural characteristics of glass. Figure 5 illustrates the diffuse reflectance (DR) spectra of the prepared glasses within wavelength range of 200–1800 nm. DR spectra demonstrate that the samples behave in the same manner as reflectivity increases at wavelengths higher than 800 nm. Additionally, different DR peaks of the samples can be seen in the UV–visible range at λ ≈ 300 to 800 nm reflects the electronic transformations of Molybdenum ions in glass matrices.

**Figure 6:**
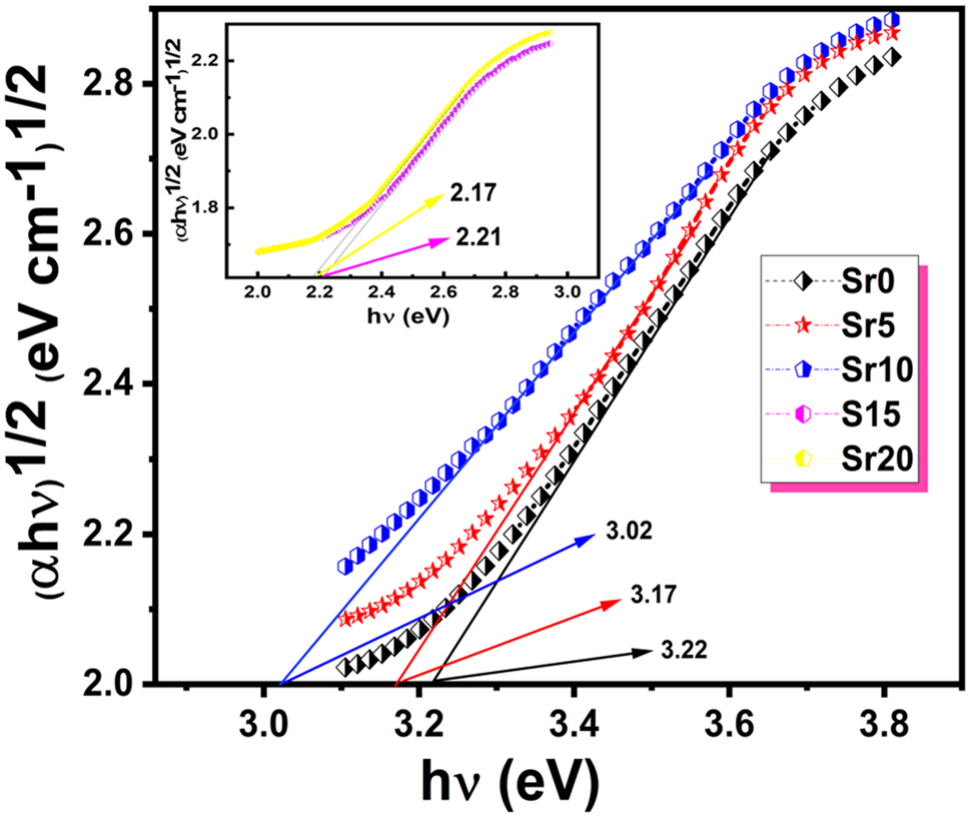
(αhν)^1/2^ as a function of (hν).

The determination of the optical transition (optical band gap) relates to the optical absorption coefficient (*α*) which was derived as aforementioned from Kubelka–Munk relation^20–23^.

9 $$\upalpha hv = C_{{1}} \left( {hv - E_{g} } \right)^{{\text{n}}}$$

where n determines the nature of electronic transition. It is well known that optical transitions take place directly (n = 1/2) or indirectly (n = 2) across the region between conduction and valence bands (optical band gap). These transitions in vitreous materials like glass, are almost indirect transitions, i.e., the effects of glass forming anions on the conduction band and cations, play a significant role in indirect transition which is always associated with absorbing or releasing phonons^49^. Figure 6 depicts (αhν)^1/2^ versus photon energy (hν) for studied samples. The linear extrapolation of the curves to the hν axis at (αhν)^1/2^ = 0 gives the value of the optical energy band gap for indirect E_opt_ transitions.

**Figure 7:**
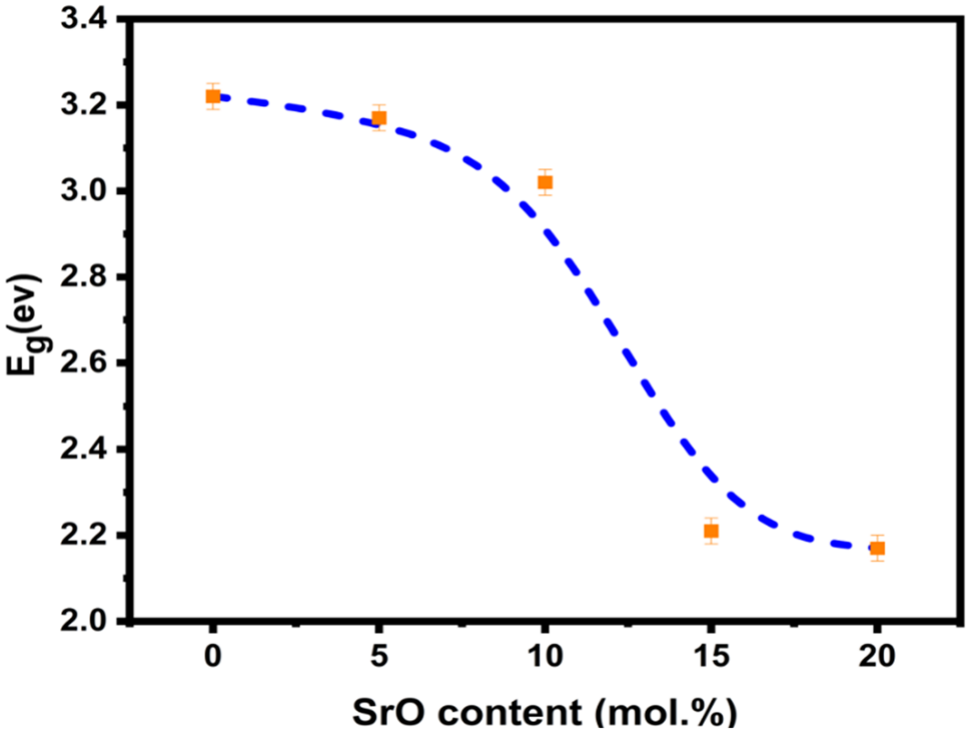
The variation of the gap energy E_g_ vs SrO content.

SrO and the base glass lattice affect the optical band gap energy in the present glass system. By increasing the SrO concentration, the indirect transition values decreased from 3.22 to 2.17 eV. It has been acknowledged that the oxygen bond strength forming the glass network directly influences the absorption edge location. These links are broken with network modifiers resulting in non-bridged oxygen. One end of the glass network structural unit is partially associated with these oxygen ions, which are partially negative in charge. Thus, the change in E_opt_ can be correlated with the change in the glass lattice, which influences cross-linking density^50^. The Fig. 7 shows how the bandgap energy E_g_ declines as the SrO content increases. This reduction may be attributed to the increase in non-bridged oxygen within the glass matrix, caused by replacing Li_2_O with SrO. These oxygens significantly contribute to the valence band due to their energy. Since non-bridging oxygens are more energetic and active than bridging oxygens, valence band energy rises and E_g_ decreases. Additionally, with a higher atomic number, coordination number, and ionic radius, the conduction band moves down more frequently. By creating non-bridging oxygen and decreasing glass structural tightness, E_g_ is lowered^38,50^.

### ESR results

**Figure 8:**
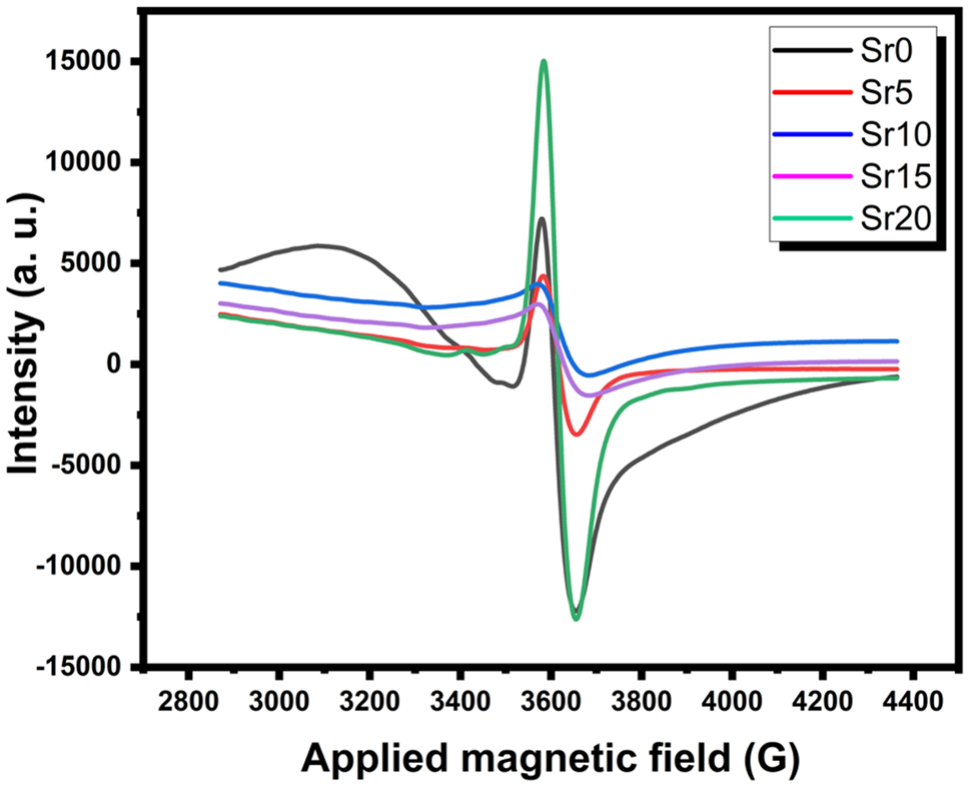
ESR spectra of the studied glasses.

ESR spectroscopy was used to confirm the status of molybdenum ions in glass samples and approve the UV–visible findings. Figure 8 displays ESR spectra at room temperature of the studied molybdenum phosphate glasses where successive portions of Li_2_O were replaced by SrO. The spectra exhibit a hyperfine structure with resonance lines have g-values, parallel at g*||*= 1.91716 and perpendicular at g⊥ = 1.93602 which are corresponding to octahedrally coordinated Mo^5+^ ions paramagnetic active species, the octahedron being trigonally distorted^51^. It is observed that with an increase in SrO content in glass composition from 0 to 20 mol%, there is no change in g-values. However, there is a noticeable increase in signal intensity. The increase may be due to the charge transition transfer between Mo^6+^ and Mo^5+^^41,51^.

### Electrical conductivity

**Figure 9:**
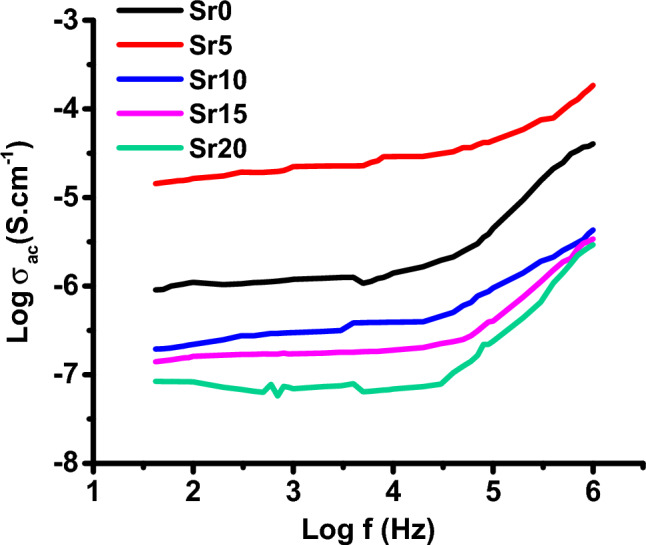
Variation of log σ_ac_ as a function of frequency measured at 298 K for glass samples.

Figure 9 illustrates the logarithmic variation of AC conductivity (log σ_ac_) as a function of frequency for all samples at room temperature (298 K) covering a frequency range from 0.042 kHz to 1 MHz. At lower frequencies, a plateau region of independent frequency appears which corresponds to DC conductivity^52^. However, in higher frequency regions, AC conductivity is more frequency-dependent. According to Fig. 9, the AC conductivity of the samples increases with frequency, implying that they are of a semiconducting character^53^. The increase in AC conductivity of the material with increasing frequency can be attributed to the release of space charges caused by a decrease in the material's barrier properties^54^. As an example, sample Sr5 exhibits an AC conductivity value of approximately 1.43 × 10^–5^ S cm^−1^ at lower frequencies, up to about 50 kHz, which then increases to 1.84 × 10^–4^ S cm^−1^ at 1 MHz, as detailed in Table 2.

**Table 2:** The DC activation energy (E_a(dc)_), the DC conductivity (σ_dc_) at room temperature (RT) and the AC conductivity (σ_ac_) at 1 MHz and at RT for all glass samples.

Sample code	σ_dc_ (S cm^−1^), RT	σ_ac_ (S cm^−1^), 1 MHz	E_a(dc)_ (eV)
Sr0	1.10 × 10^–6^	4.04 × 10^–5^	2.15
Sr5	1.64 × 10^–5^	1.84 × 10^–4^	1.64
Sr10	2.19 × 10^–7^	4.32 × 10^–6^	0.37
Sr15	1.61 × 10^–7^	3.41 × 10^–6^	0.26
Sr20	8.30 × 10^–8^	2.92 × 10^–6^	0.13

**Figure 10:**
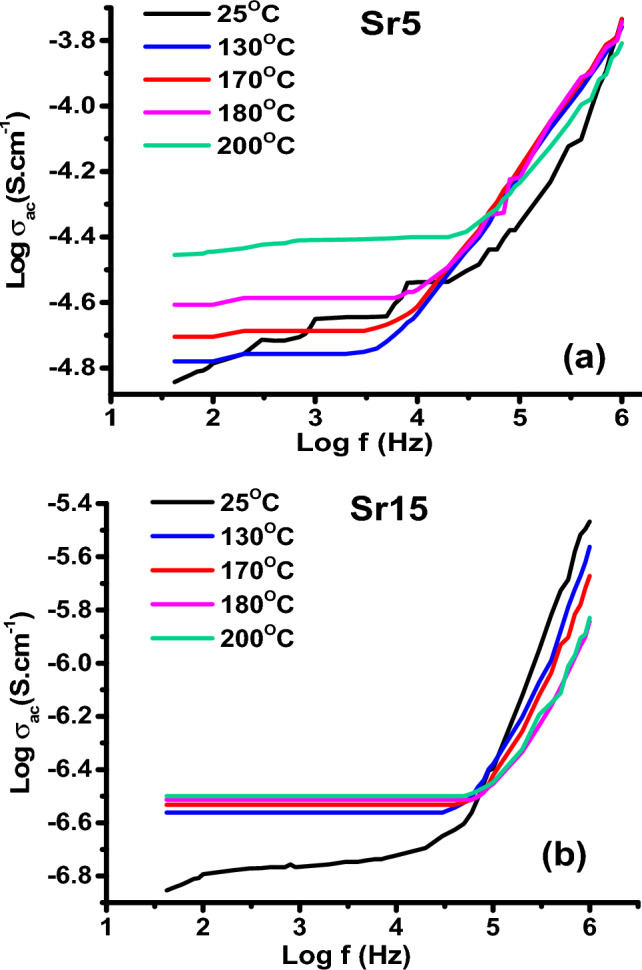
Variation of log σ_ac_ as a function of frequency for glass samples (**a**) Sr5 and (**b**) Sr15 at different temperatures.

The frequency dependence of conductivity (σ_ac_) for samples Sr5 and Sr15 (chosen as representative samples) is illustrated in Fig. 10a,b at different temperatures. It is evident from the figure that the σ_ac_ increases with increasing frequency. Furthermore, as the temperature increases the values of conductivity rise. This observation strongly suggests that the electrical conductivity of the material is a thermally activated process^55^.

**Figure 11:**
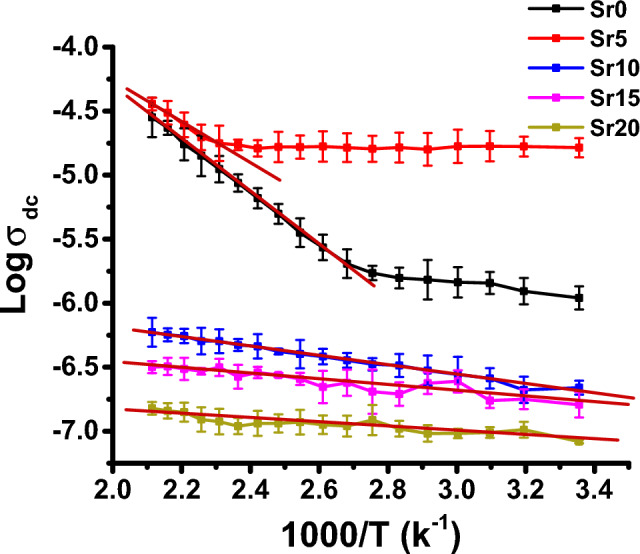
The variation of Log σ_dc_ versus 1000/T for glass samples of the composition 40 P_2_O_5_ − 20 MoO_3_ − 15 MgO-(25-x) Li_2_O-x SrO at different contents of SrO (mol%). Error bars are determined.

Figure 11 displays the temperature-dependent DC electrical conductivity of glass samples with the composition 40 P_2_O_5_ − 20 MoO_3_ − 15 MgO-(25-x) Li_2_O-x SrO, featuring varying SrO content. The electrical conductivity σ_dc_ is thermally activated process and follows the Arrhenius relation

10 $$\upsigma_{{{\text{dc}}}} = \, \upsigma_{{\text{o}}} {\text{exp }}\left( { - {\text{E}}_{{\text{a}}} /{\text{ kT}}} \right),$$

where σ_o_ is the pre-exponential factor, E_a_ is the activation energy for conduction, T is the absolute temperature and k is the Boltzmann constant. The figure reveals that the electrical conductivity (σ_dc_) of these glasses increases with temperature across the entire temperature range, indicating their semiconducting nature^56^. At the high temperature region ranging from 413 to 473 (K), the DC activation energies (E_a(dc)_) are determined from the slopes of the straight lines of the log σ_dc_ plots versus 1000/T plots and the values are listed in Table 2. The range of activation energy values falls between 0.13 and 2.15 (eV).

In addition, in Fig. 11, it can be noticed that the σ_dc_ of the samples Sr0 and Sr5 exhibits a non-linear increase with rising the measuring temperature, featuring two distinct regions. These two regions are formed by two different mechanisms. The first region, at lower temperatures ranging from room temperature up to approximately 373 K, is characterized by low activation energy. This behavior is attributed to electronic transition mechanisms, likely facilitated by the presence of molybdenum ions in multivalent states (Mo^3+^, Mo^4+^, Mo^5+^, and Mo^6+^)^57^ as confirmed by UV–visible spectra. While the second mechanism observed at higher temperatures (413–473 K) exhibits higher activation energy. This is primarily due to ionic movement mechanisms driven by the mobility of charge carriers, specifically Li^+^ ions.

Furthermore, for samples Sr10, Sr15, and Sr20, it is evident that the electrical conductivity (σdc) increases linearly with rising temperature, accompanied by relatively low activation energies ranging from 0.13 to 0.37 eV. The substantial decrease in Li^+^ concentration due to the substitution of lithium ions with alkaline-earth metal ions is expected to lead to a reduction in the number of ionic carriers, consequently resulting in decreased ionic conductivity. Moreover, the replacement of Li_2_O by SrO is found to increase the presence of non-bridging oxygen (NBO) ions, a finding confirmed by UV–visible spectra. This increase in NBO ions, in turn, enhances the mobility of charge carriers, primarily electrons. This explains the decrease of the E_a(dc)_ with the addition of SrO and suggests that the conduction in glass samples Sr10, Sr15 and Sr20 is mainly electronic.

**Figure 12:**
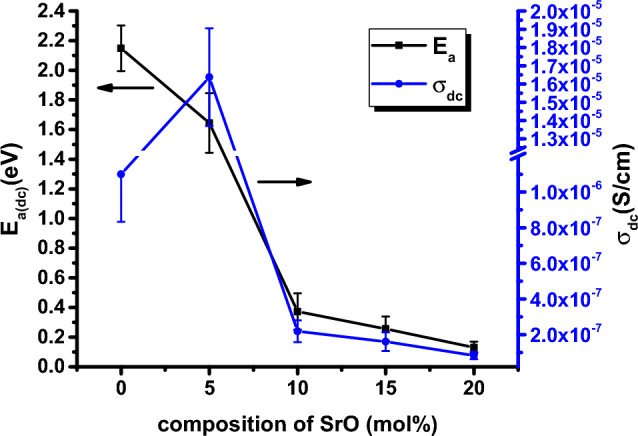
The Variation of the σ_dc_ at 298 K and high temperature activation energy, E_a(dc)_, with mole fractions of SrO for all glass samples. Error bars are determined.

Figure 12 illustrates the variations in both room temperature DC electrical conductivity (σ_dc_) and activation energies (E_a(dc)_) as a function of SrO concentration (in mol%). It is evident from this figure that σ_dc_ decreases from 1.10 × 10^–6^ to 8.31 × 10^–8^ (S/cm) as the SrO concentration increases from 0 to 20 mol%. Notably, the plot exhibits a maximum value when x = 5 mol% of SrO. To elucidate this behavior, it is worth noting that various studies suggest that alkali ions contribute more significantly to the conductivity of oxide glasses doped with alkali and alkaline earth ions, whereas alkaline earth ions have a less pronounced effect^58,59^. As a result, Li_2_O ion species exhibit greater mobility and ease of diffusion compared to SrO ion species within the glass matrix. Consequently, in the present study, the initial replacement of SrO concentration (at 5 mol%) leads to an increase in σ_dc_, reaching 1.64 × 10^–5^ S/cm, accompanied by a decrease in activation energy.

Additionally, the increase in conductivity can be attributed to structural modifications within the phosphate network. The presence of SrO is known to induce a loosening effect on the glass matrix^10,60^. Consequently, this structural change facilitates the migration of conducting ions (Li^+^ ions), which are smaller (90 pm) compared to Sr^2+^ ions (132 pm). This size disparity enhances ion mobility, resulting in increased conductivity. In this context, both Li^+^ ion concentration and the introduction of non-bridging oxygen species, a result of adding the modifier oxide SrO^9^, can influence glass conductivity. This effect is especially notable during the initial replacement with 5 mol% SrO in sample Sr5, leading to an increase in conductivity.

With further replacement of SrO, both the conductivity and activation energy decrease. This drop in conductivity is attributed to the increased presence of less mobile SrO ions at the expense of more mobile Li_2_O ions, resulting in reduced conductivity for the glass samples Sr10, Sr15, and Sr20. However, the increased number of non-bridging oxygens (NBO) with a higher concentration of SrO suggests the dominance of electronic conduction with lower activation energy. The nonlinear variation of activation energy and DC conductivity within the studied glass compositions is attributed to the occurrence of a mixed cation effect^58^.

## Conclusions

Improved molybdenum phosphate glasses with the composition 40 P_2_O_5_, 20 MoO_3_, 15 MgO, (25-x) Li_2_O, x SrO where 0 ≤ x ≤ 20 mol % were prepared via the melt-quenching technique. Increasing the strontium oxide content leads to enhanced packing of coordination polyhedra in the glass network, resulting in increased glass density and hardness. This compactness arises from Sr^2+^ ions occupying interstitial spaces within the network structure. FTIR of the base glass Sr0 displayed the characteristic absorption bands of oscillation modes of the basic building blocks of the glass lattice PO_2_, PO_3_, PO_4_^−^, MoO_4_^−^. The introduction of SrO into the glass network led to modifications as phosphate chains depolymerized, driven by the modifying behavior of Sr^2+^ ions. FTIR deconvoluted spectra confirmed structural network modifications associated with increasing SrO content, up to 20 mol%. The optical measurements revealed the presence of diverse active molybdenum ion species incorporated into the glass network. They explained the role of modifier ions in the structural network leading to an increase in non-bridging oxygen. Consequently, the bandgap energy decreased, transforming the glass from an insulator with its characteristic features into a material exhibiting semiconductor properties. Temperature dependent DC electrical conductivity was investigated. The samples' electrical conductivities increase with temperature, revealing semiconducting behavior. It was observed that the conductivity of Sr0 and Sr5 samples was the highest. This may be due to the dominance of lithium ions over their conductivity. As the concentration of strontium oxide increases beyond 5 mol%, the observed conductivity may be mainly electronic, which can be attributed to the hopping of electrons between multivalent states of molybdenum in addition to the enhanced electrons created by the increasing number of NBO ions. For samples with SrO ˃ 5 mol%, the dominance of electronic mechanism over the ionic transfer ones may present good candidates for semiconductor materials in electronic devices. In addition, the investigated glass compositions exhibited favorable optical and electrical properties, enhancing their suitability for applications in opto-electronic devices.

## Data Availability

The data that support the findings of this study are available from the corresponding author upon reasonable request.

## References

[1] Brow RK (2000). Review: the structure of simple phosphate glasses. J. Non-Cryst. Solids.

[2] Černošek Z, Holubová J, Hejda P (2019). The influence of phosphorus substitution by molybdenum on the chemical composition and certain properties of the zinc metaphosphate glass. J. Non-Cryst. Solids.

[3] Vedeanu NS, Magdas DA (2012). The influence of some transition metal ions in lead- and calcium-phosphate glasses. J. Alloys Compds.

[4] Melo BMG, Graça MPF, Prezas PR, Valente MA, Almeida AF, Freire FNA, Bih L (2016). Structural and thermal characterization of phosphate based glasses promising for hydrogen absorption. J. Non-Cryst. Solids.

[5] Abe Y, Hosono H, Ohta Y, Hench LL (1988). Protonic conduction in oxide glasses: Simple relations between electrical conductivity, activation energy, and the O–H bonding state. Phys. Rev. B.

[6] Pershina SV, Antonov BD, Leonidov II (2021). Effect of MoO_3_ on structural, thermal and transport properties of lithium phosphate glasses. J. Non-Cryst. Solids.

[7] Ouachouo L, Es-soufi H, Sayyed MI, Ghyati S, Bih L (2023). Investigation of structural, thermal, and electrical characteristics of zirconium-doped lithium-phosphate glasses. Heliyon.

[8] Jlassi I, Sdiri N, Elhouichet H (2017). Electrical conductivity and dielectric properties of MgO doped lithium phosphate glasses. J. Non-Cryst. Solids.

[9] Bouabdalli EM, El Jouad M, Gaumer N, Siniti M, Touhtouh S, Hajjaji A (2023). Structural, physical, thermal and optical spectroscopy studies of the europium doped strontium phosphate glasses. Inorganic Chem. Commun..

[10] Bouabdalli EM, El Jouad M, Touhtouh S, Hajjaji A (2023). First investigation of the effect of strontium oxide on the structure of phosphate glasses using molecular dynamics simulations. Comput. Mater. Sci..

[11] Sreeram N, Yusub S, Ramesh Babu A, Venkateswarlu M, Krishna Priya G, Aruna V, Koutavarapu R (2023). Ascendancy of Cr_2_O_3_ on morphology, spectroscopic and dielectric properties of GeO_2_–Li_2_O–P_2_O_5_–MgO glasses. Mater. Chem. Phys..

[12] Bih L, Nadiri A, El Amraoui Y (2005). Investigation of the physico-chemical properties of NaPO_3_-MoO_3_ glasses. J. Phys. IV France.

[13] Santagneli SH, de Araujo CC, Strojek W, Eckert H, Poirier G, Ribeiro SJL, Messaddeq Y (2007). Structural studies of NaPO_3_−MoO_3_ glasses by solid-state nuclear magnetic resonance and Raman spectroscopy. J. Phys. Chem. B.

[14] Bih L, El Omari M, Reau J, Nadiri A, Yacoubi A, Haddad M (2001). Electrical properties of glasses in the Na_2_O–MoO_3_–P_2_O_5_ system. Mater. Lett..

[15] Chowdari BVR, Tan KL, Chia WT, Gopalakrishnan R (1991). Thermal, physical, electrical and XPS studies of the Li_2_O:P_2_O_5_: MoO_3_ glass system. J. Non-Cryst. Solids.

[16] Lakshmana Rao B, Jyothi Raju K, Prasad SVGVA (2018). Optical absorption spectra of PbO-Sc_2_O_3_-P_2_O_5_ glasses doped with molybdenum ions. Mater. Today Proc..

[17] Bih L, El Omari M, Reau JM, Haddad M, Boudlich D, Yacoubi AA (2000). Electronic and ionic conductivity of glasses inside the Li_2_O–MoO_3_–P_2_O_5_ system. Solid State Ion..

[18] Mogus-Milankovic A, Santic A, Karabulut M, Day DE (2003). Study of electrical properties of MoO_3_–Fe_2_O_3_–P_2_O_5_ and SrO–Fe_2_O_3_–P_2_O_5_ glasses by impedance spectroscopy. J. Non-Cryst. Solids.

[19] Mugoni C, Montorsi M, Siligardi C, Jain H (2014). Electrical conductivity of copper lithium phosphate glasses. J. Non-Cryst. Solids.

[20] Kubelka P (1948). New contribution of optics of intensely light materials. JOSA..

[21] Brow RK (2000). Review: The structure of simple phosphate glasses. J. Non-Cryst. Solids.

[22] Tauc J, Menth A (1972). Localized states in the gap. J. Non-Cryst. Solids.

[23] Lia H, Yia J, Qin Z, Sun Z, Xu Y, Wang Ch, Zhao F, Hao Y, Liang X (2019). Structures, thermal expansion, chemical stability and crystallization behavior of phosphate-based glasses by influence of rare earth. J. Non-Cryst. Solids.

[24] Shukla N, Pathak HP, Rao V, Dwivedi DKAC (2016). conductivity and dielectric properties of Se_90_Cd_6_Sb_4_ glassy alloy. Chalcogenide Lett..

[25] Cullity BD (1978). Elements of Diffraction Quasi-Optics.

[26] Jha PK, Pandey OP, Singh K (2015). Structure and crystallization kinetics of Li_2_O modified sodium-phosphate glasses. J. Mol. Struct..

[27] Du J, Xiang Y (2012). Effect of strontium substitution on the structure. Ionic diffusion and dynamic properties of 45S5 bioactive glasses. J. Non-Cryst. Solids.

[28] Hill RG, Stamboulis A, Law RV, Clifford A, Towler MR, Crowley C (2004). The influence of strontium substitution in flouroapatite glasses and glass-ceramics. J. Non-Cryst. Solids.

[29] Marzouk MA, Hamdy YM, ElBatal HA (2017). Photoluminescence and spectral performance of manganese ions in zinc phosphate and barium phosphate host glasses. J. Non-Cryst. Solids.

[30] Marzouk S, Abo-Naf SM, Hammam M, El-Gendy YA, Hassan NS (2011). FTIR spectra and optical properties of molybdenum phosphate glasses. J. Appl. Sci. Res..

[31] Ouis MA, Gamal AA (2021). Role of silver ions in Na_2_O-CaF_2_-P_2_O_5_ host glass and its corresponding glass-ceramic: Searching for antibacterial behaviour-supplemented by spectral, optical, FTIR and SEM investigations. Egypt. J. Chem..

[32] Misbah MH, El-Kemary M, Ramadan R (2021). Effect of Mg^2+^ coordination on the structural and optical properties of iron magnesium phosphate glasses. J. Non-Cryst. Solids.

[33] Ouis MA, Azooz MA, ElBatal HA (2018). Optical and infrared spectral investigations of cadmium zinc phosphate glasses doped with WO_3_ or MoO_3_ before and after subjecting to gamma irradiation. J. Non-Cryst. Solids.

[34] El-Batal AM, Saeed A, Hendawy N, El-Okr MM, El-Mansy MK (2021). Influence of Mo or/and Co ions on the structural and optical properties of phosphate zinc lithium glasses. J. Non-Cryst. Solids.

[35] Sułowska J, Wacławska I, Olejniczak Z (2013). Structural studies of copper-containing multicomponent glasses from the SiO_2_–P_2_O_5_–K_2_O–CaO–MgO system. Vib. Spectrosc..

[36] Omrani RO, Kaoutar A, El Jazouli A, Krimi S, Khattech I, Jemal M, Videau JJ, Couzi M (2015). Structural and thermochemical properties of sodium magnesium phosphate glasses. J. Alloys Compds..

[37] Farouk M, Samir A, El Okr M (2018). Effect of alkaline earth modifier on the optical and structural properties of Cu^2+^ doped phosphate glasses as a bandpass filter. Phys. B Phys. Condens. Matter.

[38] Sastry SS, Rao BRV (2014). spectroscopic studies of copper doped alkaline earth lead zinc phosphate glasses. Phys. B.

[39] ElBatal FH, Abdelghany AM, Elwan RL (2011). Structural characterization of gamma irradiated lithium phosphate glasses containing variable amounts of molybdenum. J. Mol. Struct..

[40] Ivascu C, Timar Gabor A, Cozar O, Daraban L, Ardelean I (2010). FT-IR, Raman and thermoluminescence investigation of P_2_O_5_–BaO–Li_2_O glass system. J. Mol. Struct..

[41] Turky GM, Fayad AM, El-Bassyouni GT, Abdel-Baki M (2021). Dielectric and electrical properties of MoO_3_-doped borophosphate glass: Dielectric spectroscopy investigations. J. Mater. Sci. Mater. Electron..

[42] Weyl, W. A. *Coloured Glasses, Monograph Reprinted by Dawson’s of Pall Mall, London* (1959).

[43] ElBatal FH, Abo Naf SM, Marzouk SY (2011). Gamma ray interactions with MoO_3_-doped lead phosphate glasses. Philos. Mag..

[44] Rao BL, Raju KJ, Prasad SVGVA (2018). Optical absorption spectra of PbO-Sc_2_O_3_-P_2_O_5_ glasses doped with molybdenum ions. Mater. Today Proc..

[45] Es-soufi H, Bih L (2021). Effect of TiO_2_ on the chemical durability and optical properties of Mo-based phosphate glasses. J. Non-Cryst. Solids.

[46] Ehrt D, Ebeling P, Natura U (2000). UV Transmission and radiation-induced defects in phosphate and fluoride–phosphate glasses. J. Non-Cryst. Solids.

[47] Urbach F (1953). The long-wavelength edge of photographic sensitivity and of the electronic absorption of solids. Phys. Rev..

[48] Toloman D, Ciceo-Lucacel R, Magdas DA, Regos A, Biris AR, Leostean C, Ardelean I (2013). The modifier / former role of MoO_3_ in some calcium-phosphate glasses. J. Alloys Compds..

[49] Fayad AM, Shaaban KhS, Abd-Allah WM, Ouis M (2020). Structural and optical study of CoO doping in borophosphate host glass and effect of gamma irradiation. J. Inorganic Organomet. Polym. Mater..

[50] Abdel-Baki M, Mostaf AM, Fayad AM, El-Bassyouni GT, Turky GM (2023). Improving the optical, electrical, and dielectric characteristics of MgO doped borate glass for optoelectronic applications. J. Appl. Phys..

[51] Abo-Naf SM, Elwan RL, ElBatal HA (2018). Photoluminescence, optical properties, thermal behavior and nanocrystallization of molybdenum-doped borobismuthate glasses. J. Mater. Sci. Mater. Electron..

[52] Zangina T, Hassan J, Matori KA, Azis RS, Ahmadu U, Alex S (2016). Sintering behavior, ac conductivity and dielectric relaxation of Li_1.3_Ti_1.7_Al_0.3_(PO_4_)_3_ NASICON compound. Results Phys..

[53] Morsi RMM, Basha MAF, Morsi MM (2016). Synthesis and physical characterization of amorphous silicates in the system SiO_2_-Na_2_O–RO (R = Zn, Pb or Cd). J. Non-Cryst. Solids.

[54] Gomaa MM, Abo-Mosallam HA, Darwish H (2009). Electrical and mechanical properties of alkali barium titanium alumino borosilicate glass-ceramics containing strontium or magnesium. J. Mater. Sci. Mater. Electron..

[55] Song CH, Choi HW, Kim M, Jin GY, Yang YS (2007). Electrical relaxations of amorphous xKNbO_3_ − (1–x)SiO_2_ (x = 0.33, 0.5, 0.67, 0.8). J. Korean Phys. Soc..

[56] Banagar AV, Kumar MB, Nagaraja N, Tipperudra A, Jakati S (2020). DC electrical conduction in strontium vanadium borate glasses. Mater. Sci..

[57] Elalaily NA, Zahran AH, Sallam OI, EzzEldin FM (2019). Structure and electrical conductivity of ɤ-irradiated lead–phosphate glass containing MoO_3_. Appl. Phys. A.

[58] Thipperudra A, Nagaraja N, Prashanth Kumar M, Arun Kumar B (2020). AC conductivity and dielectrics studies in potassium, strontium doped borophosphate glasses. Int. J. Innov. Res. Multidiscip. Field.

[59] Bahgat AA, Abou-Zeid YM (2001). Mixed alkali effect in the K_2_O–Na_2_O–TeO_2_ glass system. Phys. Chem. Glasses.

[60] Mroczkowska M, Nowinski JL, Zukowska GZ, Mroczkowska A, Garbarczyk JE, Wasiucionek M, Gierlotka ST (2007). Micro Raman, FT-IR / PAS, XRD and SEM studies on glassy   and partly crystalline silver phosphate ionic conductors. J. Power Source.

